# Non-inferiority trials: are they inferior? A systematic review of reporting in major medical journals

**DOI:** 10.1136/bmjopen-2016-012594

**Published:** 2016-10-07

**Authors:** Sunita Rehal, Tim P Morris, Katherine Fielding, James R Carpenter, Patrick P J Phillips

**Affiliations:** 1 MRC Clinical Trials Unit at UCL, Institute of Clinical Trials and Methodology, London, UK; 2 MRC Clinical Trials Unit at UCL, London Hub for Trials Methodology Research, London, UK; 3 MRC Tropical Epidemiology Group, Department of Infectious Disease Epidemiology, London School of Hygiene & Tropical Medicine, London, UK; 4 Department of Medical Statistics, London School of Hygiene & Tropical Medicine, London, UK

**Keywords:** non-inferiority, systematic review, randomised controlled clinical trials, clinical trial

## Abstract

**Objective:**

To assess the adequacy of reporting of non-inferiority trials alongside the consistency and utility of current recommended analyses and guidelines.

**Design:**

Review of randomised clinical trials that used a non-inferiority design published between January 2010 and May 2015 in medical journals that had an impact factor >10 (*JAMA Internal Medicine*, *Archives Internal Medicine*, *PLOS Medicine*, *Annals of Internal Medicine*, *BMJ*, *JAMA*, *Lancet* and *New England Journal of Medicine*).

**Data sources:**

Ovid (MEDLINE).

**Methods:**

We searched for non-inferiority trials and assessed the following: choice of non-inferiority margin and justification of margin; power and significance level for sample size; patient population used and how this was defined; any missing data methods used and assumptions declared and any sensitivity analyses used.

**Results:**

A total of 168 trial publications were included. Most trials concluded non-inferiority (132; 79%). The non-inferiority margin was reported for 98% (164), but less than half reported any justification for the margin (77; 46%). While most chose two different analyses (91; 54%) the most common being intention-to-treat (ITT) or modified ITT and per-protocol, a large number of articles only chose to conduct and report one analysis (65; 39%), most commonly the ITT analysis. There was lack of clarity or inconsistency between the type I error rate and corresponding CIs for 73 (43%) articles. Missing data were rarely considered with (99; 59%) not declaring whether imputation techniques were used.

**Conclusions:**

Reporting and conduct of non-inferiority trials is inconsistent and does not follow the recommendations in available statistical guidelines, which are not wholly consistent themselves. Authors should clearly describe the methods used and provide clear descriptions of and justifications for their design and primary analysis. Failure to do this risks misleading conclusions being drawn, with consequent effects on clinical practice.

**Strengths and limitations of this study**

- This research clearly demonstrates the inconsistency in recommendations for non-inferiority trials provided by guidelines for researchers and this is reflected within this review.
- It highlights missing data and sensitivity analyses in the context of non-inferiority trials.
- It provides recommendations using examples for researchers using the non-inferiority design.
- Justification of the choice of the margin was recorded as such if any attempt was made to do so, and so one could argue that inadequate attempts were counted as a ‘justification’; however, there was good agreement between reviewers when independently assessed.
- Only one reviewer extracted information from all articles and therefore assessments may be subjective. However, there was good agreement when a random 5% of papers were independently assessed.

## Introduction

Non-inferiority trials assess whether a new intervention is not much worse when compared to a standard treatment or care. These trials answer whether we are willing to accept a new intervention that may be clinically worse, yet still be beneficial for patients while having another advantage, such as less-intensive treatment, lower cost or fewer side effects.^1^ Non-inferiority and equivalence are sometimes, mistakenly, used interchangeably. Equivalence trials are designed to show that a new intervention performs not much worse and not much better than a standard intervention. Both trial designs are different to superiority trials, which aim to show that a new intervention performs better when compared to a control.

Poor trial quality can bias trial results towards concluding no difference between treatments.^2^ This creates more challenges in non-inferiority trials than superiority trials as such bias can produce false-positive results for non-inferiority.^3–5^ The increasing use of this design^6–8^ means that it is even more important for trialists to understand the issues around the quality in the design and analysis of non-inferiority trials.

There are several guidelines available to aid researchers using a non-inferiority design, where various considerations of the design are explained and discussed (table 1).

1. The CONSORT extension statements^1^ ^9^ focus on the reporting of non-inferiority trials, with the most recent 2012 statement being an elaboration of the 2006 statement.
2. The draft FDA 2010^2^ document focuses on all aspects and issues relative to non-inferiority trials and gives general guidance.
3. The EMEA 2000 guideline^10^ discusses switching between non-inferiority and superiority designs and the EMEA 2006^11^ guideline discusses the choice of the non-inferiority margin, taking into account two-arm and three-arm trials.
4. The ICH E9 and E10 guidelines^12^ ^13^ are general statistical guidance documents addressing issues for all clinical trials and designs.
5. SPIRIT^14^ is a guidance document for protocols for all trial designs and includes discussions of recently developed methodology.

**Table 1:** Summary of guidelines

	Justification of margin	Who is included in analysis	CI	Missing data	Sensitivity analyses
CONSORT 2006^1^	‘Margin should be specified and preferably justified on clinical grounds’	‘Non-ITT analyses might be desirable as a protection from ITTs increase in type I error. There is greater confidence in results when the conclusions are consistent’. ITT: ‘Analysing all patients within their randomised groups, regardless of whether they completed allocated treatment is recommended’ *PP:* ‘Alternative analyses that exclude patients not taking allocated treatment or otherwise not protocol-adherent could bias the trial in either direction. The terms on-treatment or PP analysis are often used but may be inadequately defined’.	‘Many non-inferiority trials based their interpretation on the upper limit of a one-sided 97.5% CI, which is the same as the upper limit of a two-sided 95% CI’. ‘Although one-sided and two-sided CIs allow for inferences about non-inferiority, we suggest that two-sided CIs are appropriate in most non-inferiority trials. If a one-sided 5% significance level is deemed acceptable for the non-inferiority hypothesis test (a decision open to question), a 90% two-sided CI could then be used’.		
CONSORT 2012^9^		‘Should be indicated if conclusions are related to PP analysis, ITT analysis or both and if the conclusions are stable between them’.	‘The two-sided CI provides additional information, in particular for the situation in which the new treatment is superior to the reference treatment’		Sensitivity analysis is discussed through an example: ‘Study endpoints were analysed primarily for the PP population and repeated, for sensitivity reasons, for the ITT population’.
Draft FDA 2010^2^	‘Whether M1 (the effect of the active control arm relative to placebo) is based on a single study or multiple studies, the observed (if there were multiple studies) or anticipated (if there is only one study) statistical variation of the treatment effect size should contribute to the ultimate choice of M1, as should any concerns about constancy. The selection of M2 (the largest clinically acceptable difference of the test treatment compared to the active control) is then based on clinical judgment regarding how much of the M1 active comparator treatment effect can be lost. The exercise of clinical judgment for the determination of M2 should be applied after the determination of M1 has been made based on the historical data and subsequent analysis’	‘It is therefore important to conduct both ITT and “as-treated” analyses in non-inferiority studies’. ITT: ‘preserve the principle that all patients are analysed according to the treatment to which they have been randomised even if they do not receive it’	‘Typically, the one-sided type I error is set at 0.025, by asking that the upper bound of the 95% CI for control treat be less than the NI margin. If multiple studies provide very homogeneous results for one or more important endpoints, it may be possible to use the 90% lower bound rather than the 95% lower bound of the CI to determine the active control effect size’	‘Poor quality can reduce the drug's effect size and undermine the assumption of the effect size of the control agent, giving the study a “bias towards the null”’.	
ICH E9^12^	‘This margin is the largest difference that can be judged as being clinically acceptable’	‘In confirmatory trials, it is usually appropriate to plan to conduct an analysis of the full analysis set and a PP analysis. In an equivalence or non-inferiority trial, use of the full analysis set is generally not conservative and its role should be considered very carefully’. ITT: ‘participants allocated to a treatment group should be followed up, assessed and analysed as members of that group irrespective of their compliance to the planned course of treatment’. *Full analysis set:* ‘The set of participants that is as close as possible to the ideal implied by the ITT principle. It is derived from the set of all randomised participants by minimal and justified elimination of participants’. *PP:* ‘The set of data generated by the subset of participants who complied with the protocol sufficiently to ensure that these data would be likely to exhibit the effects of treatment, according to the underlying scientific model. Compliance covers such considerations as exposure to treatment, availability of measurements and absence of major protocol violations’.	‘For non-inferiority trials, a one-sided interval should be used. The choice of type I error should be a consideration separate from the use of a one-sided or two-sided procedure’.	‘Imputation techniques, ranging from LOCF to the use of complex mathematical models, may be used to compensate for missing data’	‘An investigation should be made concerning the sensitivity of the results of analysis to the method of handling missing values, especially if the number of missing values is substantial’.
ICH E10^13^	‘The determination of the margin in a non-inferiority trial is based on statistical reasoning and clinical judgment’				
SPIRIT^14^		Use an example where ‘non-inferiority would be claimed if ITT and PP analyses show conclusions of NI’. ITT: ‘To preserve the unique benefit of randomisation as a mechanism to avoid selection bias, an “as randomised’ analysis retains participants in the group to which they were originally allocated. To prevent attrition bias, outcome data obtained from all participants are included in the data analysis, regardless of protocol adherence’. *PP and mITT:* ‘Some trialists use other types of data analyses (commonly labelled as “mITT” or “PP”) that exclude data from certain participants—such as those who are found to be ineligible after randomisation or who deviate from the intervention or follow-up protocols. This exclusion of data from protocol non-adherers can introduce bias, particularly if the frequency of and the reasons for non-adherence vary between the study groups’.		‘Multiple imputation can be used to handle missing data although relies on untestable assumptions’	‘Sensitivity analyses are highly recommended to assess the robustness of trial results under different methods of handling missing data’
EMEA 2006^11^	‘The choice of delta must always be justified on clinical and statistical grounds’		‘A two-sided 95% CI (or one-sided 97.5% CI) is constructed. The interval should lie entirely on the positive side of the margin. Statistical significance is generally assessed using the two-sided 0.05 level of significance (or one-sided 0.025)’		
EMEA 2000^10^		‘ITT and PP analyses have equal importance and their use should lead to similar conclusions for robust interpretation’	‘A two-sided CI should lie entirely to the right of delta. If one-sided confidence is used then 97.5% should be used’		‘It will be necessary to pay particular attention to demonstrating the sensitivity of the trial by showing similar results for the full analysis set and PP analysis set’

ITT, intention to treat; LOCF, last observation carried forward; mITT, modified intention to treat; NI, non-inferiority; PP, per-protocol.

There is some inconsistency between these guidelines regarding the conduct of non-inferiority trials (table 1) that may adversely affect the overall quality and reporting of non-inferiority trials. Non-inferiority trials require more care around certain issues, and so clear guidance on how to design and analyse these trials are necessary. Some of these issues that can influence inferences made about non-inferiority are outlined below.

First, the non-inferiority margin—the value that allows for a new treatment to be ‘acceptably worse’^1^—is used as a reference for conclusions about non-inferiority. It is recommended by all guidelines that this margin is chosen on a clinical basis, meaning the maximum clinically acceptable extent to which a new drug can be less effective than the standard of care and still show evidence of an effect.^15^ However, it is unclear whether statistical considerations should also affect the choice of an appropriate margin, as recommended by the Draft FDA 2010, ICH E10 and EMEA 2006 guidelines^2^ ^11^ ^13^ (table 1). Ignoring statistical evidence from meta-analyses or systematic reviews could have the potential for researchers to choose an unrealistic margin.

Second, it is important to choose who is included in analyses for non-inferiority trials. The intention-to-treat (ITT) analysis (includes all randomised patients irrespective of postrandomisation occurrences) is preferred for superiority trials as it is likely to lead to a treatment effect closer to having no effect and so is conservative.^16^ For non-inferiority trials, the ITT analysis can bias towards the null, which may lead to false claims of non-inferiority.^17^ The alternative per-protocol (PP) analysis is often considered instead. However, given that the PP analysis allows for the exclusion of patients, it fails to preserve a balance of patient numbers between treatment arms (ie, randomisation) that ITT analysis does and can cause bias in either direction, depending on who the analysis excludes.^18^ Guidelines often recommend performing the ITT and PP analyses, although definitions are inconsistent (table 1). In particular, the CONSORT 2006 guidelines describe the PP analysis as excluding patients not taking allocated treatment or otherwise not protocol-adherent,^1^ whereas the ICH E9 guidelines state that the PP analysis is a “subset of patients who complied sufficiently with the protocol, such as exposure to treatment, availability of measures and absence of major protocol violations.”^19^ These obscure definitions could lead researchers to arbitrarily exclude patients from analyses. The draft FDA guidelines recommend researchers to use an ITT and as-treated analysis, although it is unclear what is meant by ‘as-treated’ as this is not defined within the guidelines. Other frequently used classifications such as modified ITT (mITT), which aims to contain ‘justifiable’ exclusions (eg, patients who never had the disease of interest) from the ITT analysis, are also defined inconsistently.^20^ Third, while two-sided 95% CIs are widely used for superiority trials, there is some inconsistent advice as whether to calculate 90% or 95% CIs for non-inferiority trials and whether these should be presented as one-sided or two-sided intervals (table 1).

Fourth, the handling of missing data is generally discussed for all trials but rarely in the specific context of non-inferiority trials. Methods recommended to handle missing data vary between guidelines. The ICH E9 guidelines recommend using a last observation carried forward imputation method,^19^ and the more recent SPIRIT guidelines recommend multiple imputation, but caution the reader that it relies on untestable assumptions^14^ (table 1). Methods to handle missing data often contain untestable assumptions and so, sensitivity analyses are essential to test the robustness of conclusions under different assumptions.^12^ However, it is unclear what sensitivity analyses are appropriate for non-inferiority trials.

Given the inconsistency between guidelines, we hypothesised that poor conduct and reporting would be associated with demonstrating non-inferiority. This review investigates the quality of conduct and reporting for non-inferiority trials in a selection of high-impact journals over a 5-year period. We also provide recommendations to aid trialists who may consider a non-inferiority design.

## Methods

Medical journals (general and internal medicine) with an impact factor >10 according to the ISI web of knowledge^21^ were included in the review (correct at time of search on 31 May 2015), the rationale being that articles published in these journals are likely to have the highest influence on clinical practice and be the most rigorously conducted and reported due to the thorough editorial process. We searched Ovid (MEDLINE) using the search terms ‘noninferior’, ‘non-inferior’, ‘noninferiority’ and ‘non-inferiority’ in titles and abstracts between 1 January 2010 and 31 May 2015 in *New England Journal of Medicine* (*NEJM*), *Lancet*, *JAMA*, *British Medical Journal*, *Annals of Internal Medicine*, *PLOS Medicine* and *Archives of Internal Medicine* (descending impact order). From 2013, *Archives of Internal Medicine* was renamed *JAMA Internal Medicine*, and, therefore, both journals have been included in this review. All journals refer authors to the CONSORT statement and checklist when reporting. Eligibility of articles was assessed via abstracts by two reviewers (SR and TPM). Articles included were non-inferiority randomised controlled clinical trials. Articles were excluded if the primary analysis was not for non-inferiority. Systematic reviews, meta-analyses and commentaries were also excluded. Few trials were designed and analysed using Bayesian methods and were therefore excluded for consistent comparability in frequentist methods.

Before performing the review, a data extraction form was developed to extract information from articles. Information extracted was with regard to the primary outcome. The form was standardised to collect information on the year of publication, non-inferiority margin (and how the margin was justified), randomisation, type of intervention, disease area, sample size, analyses performed (how this was defined and what was classed as primary/secondary), primary outcome, p values (and whether this was for a superiority hypothesis), significance level of CIs (and whether both bounds were reported), imputation techniques for missing data, sensitivity analyses, conclusions of non-inferiority and whether a test for superiority was prespecified. Justifications for the choice of the non-inferiority margin were reviewed by two reviewers (SR and PPJP). See online supplementary material for further details on methods.

10.1136/bmjopen-2016-012594.supp1 Supplementary data

A quality grading system was developed based on whether the margin was justified (yes vs no/poor), how many analyses were performed on the primary outcome (<2 vs ≥2) and whether the type I error rate was consistent with the significance level of the CI (yes vs no/unclear). Articles were classed as ‘excellent’ if all these criteria were fulfilled and were classed as ‘poor’ if none was fulfilled. Articles which satisfied one criterion were classed as ‘fair’ and articles that provided two of the three criteria were classified as ‘good’. The results of this grading were compared to inferences on non-inferiority to assess if the quality of reporting was associated with concluding non-inferiority at the 5% significance level.

Additional published online supplementary material was accessed only if it specifically referred to the information we were extracting within articles. As a substudy, all statistical methods, outcomes and sample sizes from protocols and/or online supplementary material were reviewed from *NEJM* as the journal is known to specifically request and publish protocols and statistical analysis plans alongside accepted publications.

Assessments were carried out by one reviewer (SR), with a random selection of 5% independently reviewed (PPJP). Any assessments that required a second opinion were independently reviewed (TPM). Any discrepancies were resolved by discussion between reviewers.

All analyses were conducted using Stata V.14.

## Results

**Figure 1:**
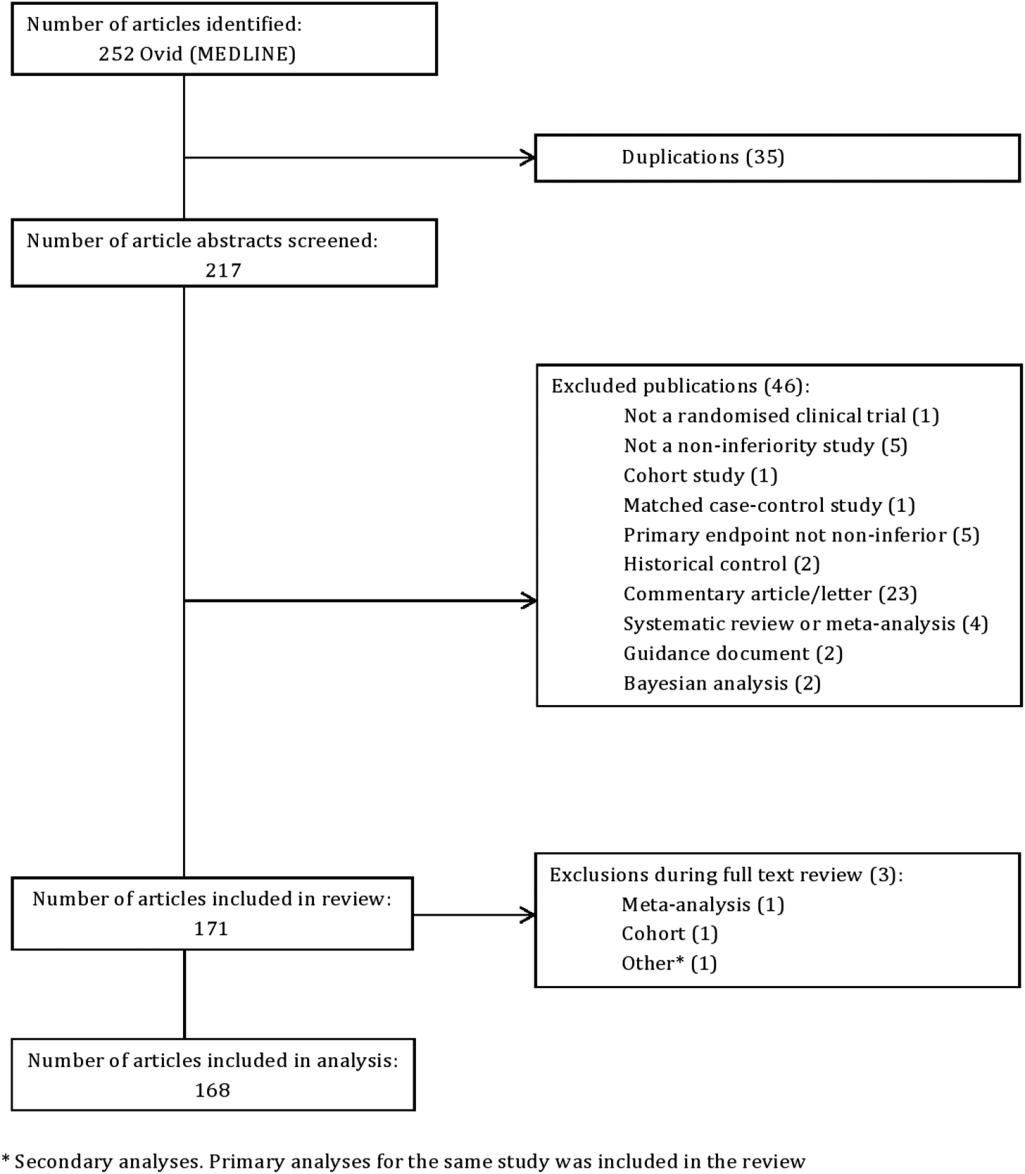
Flow chart of eligibility of articles.

Our search found 252 articles. After duplicate publications were removed, 217 were screened for eligibility using their titles and abstracts. A total of 46 articles were excluded leaving 171 articles to be reviewed. A further three articles were excluded during the full-text review leaving 168 articles (figure 1).

General characteristics of the included studies are summarised in table 2.

**Table 2:** General characteristics

	All articles (n=168)	Including *NEJM* protocols (n=61)
Characteristics	n (%)	n (%)
Journal		
*NEJM*	61 (36)	61
*Lancet*	64 (38)	
*JAMA*	19 (11)	
*BMJ*	8 (5)	
*Annals of Internal Medicine*	5 (2)	
*PLoS Medicine*	7 (4)	
*Archives of Internal Medicine*	2 (1)	
*JAMA of Internal Medicine*	2 (1)	
Year of publication		
2010	26 (15)	9 (15)
2011	27 (16)	9 (15)
2012	29 (17)	8 (13)
2013	39 (23)	19 (31)
2014	27 (16)	10 (16)
2015	20 (12)	6 (10)
Type of intervention		
Drug	112 (67)	44 (72)
Surgery	22 (13)	7 (11)
Other	34 (20)	10 (16)
Randomisation		
Patient	163 (97)	59 (97)
Cluster	5 (3)	2 (3)
Power		
80%	6 (36)	19 (31)
85%	11 (7)	5 (8)
90%	65 (39)	26 (43)
71–99% (excluding the above)	21 (12)	11 (18)
Not reported/unclear	10 (6)	0
Composite outcome		
Yes	78 (46)	37 (61)
No	90 (54)	24 (39)
Disease		
Heart disease	30 (18)	13 (21)
Blood disorder	19 (11)	6 (10)
Cancer	16 (10)	8 (13)
Diabetes	11 (7)	2 (3)
Thromboembolism	6 (4)	6 (10)
Skin infection (non-contagious)	3 (2)	2 (3)
Urinary tract infection	3 (2)	0
Arthritis	3 (2)	1 (2)
Opthomology	3 (2)	1 (2)
Pneumonia	3 (2)	1 (2)
Complications in pregnancy	3 (2)	0
Stroke	3 (2)	2 (3)
Testing method	3 (2)	1 (2)
Appendicitis	2 (1)	1 (2)
Depression	2 (1)	0
Other non-infectious disease	18 (11)	7 (11)
HIV	18 (11)	2 (3)
Tuberculosis	6 (4)	4 (7)
Malaria	4 (2)	1 (2)
Skin infection (contagious)	2 (1)	0
Hepatitis C	2 (1)	2 (3)
Other infectious disease	8 (5)	1 (2)

### Margin

The non-inferiority margin was specified in 164 (98%) articles and was justified in less than half of articles 76 (45%). The most common justification was on a clinical basis (29 (17%)), which was often worded ambiguously and with little detail. A total of 14 (8%) used previous findings from past trials or statistical reviews to justify the choice of the margin (table 3).

**Table 3:** Justification of choice of margin, total number of patient populations considered for analyses and patient population included in the analysis

	All articles (N=168)	Including *NEJM* protocols (N=61)
	n (%)	n (%)
Justification of NI margin		
Made no attempt for justification	90 (54)	22 (36)
Clinical basis. No evidence for consultation with external expert group, and no reference to previous trials of the control arm	32 (19)	11 (18)
Preservation of treatment effect based on estimates of control arm effect from previous trials	13 (8)	14 (23)
Expert group external to the authors. No reference to previous trials of the control arm	6 (4)	3 (5)
The same margin as was used in other similar trials	5 (3	2 (3)
10–12% recommended by disease-specific FDA guidelines	4 (2)	1 (2)
General comment that margin was decided according to FDA/regulatory guidance	4 (2)	0
Clinical basis and based on previous similar trial. No evidence for consultation with external expert group, and no reference to previous trials of the control arm	3 (2)	0
Based on registry/development programme	0	2 (3)
Other*	11 (7)	6 (10)
Number of analyses		
One	65 (39)	15 (25)
Two	91 (54)	38 (62)
Three	10 (6)	7 (11)
Not defined	2 (1)	1 (2)
Analysis		
ITT	129 (77)	44 (72)
PP	90 (54)	35 (57)
mITT	34 (20)	17 (28)
As-treated	4 (2)	6 (10)
Other	20 (12)	10 (16)
Unclear	2 (1)	2 (3)

*See online supplementary material.

ITT, intention to treat; mITT, modified intention to treat; PP, per-protocol.

### Patients included in analysis

Over a third of articles 65 (39%) declared only one analysis (table 3 and see online supplementary table S1a). The majority of trials classed ITT analysis as primary and PP analyses as secondary (see online supplementary figure S1a). PP analyses were performed in 90 (54%) trials; of which, 11 (12%) did not define what was meant by ‘PP’ (table 3 and see online supplementary table S1b). Definitions of the PP population contained various exclusions, mostly regarding errors in randomised treatment or treatment received.

### Type I error rate

Consistency between the type I error rate and CIs reported was moderate at 95 (57%) (table 4). Most articles, 69 (41%), used a one-sided 2.5% or (numerically equivalent) two-sided 5% significance level (table 5) and some used a one-sided 5% significance level, 46 (27%). The majority of articles presented two-sided CIs (147; 88%) and 19 (11%) articles presented one-sided CIs. Most two-sided CIs were at the 95% significance level: 125 (74%).

**Table 4:** Consistency of type I error rate with significance levels of CIs over year of publication

	Year of publication						
	2010	2011	2012	2013	2014	2015	Total
All articles (N=168)							
Yes	11 (42%)	15 (56%)	15 (52%)	24 (62%)	19 (70%)	11 (55%)	95 (57%)
No	5 (19%)	4 (15%)	4 (14%)	5 (13%)	5 (19%)	3 (15%)	26 (15%)
Not reported	10 (38%)	8 (30%)	10 (34%)	10 (26%)	3 (11%)	6 (30%)	47 (28%)
*NEJM* subgroup (N=61)							
Yes	7 (78%)	6 (67%)	5 (63%)	14 (74%)	8 (80%)	4 (67%)	44 (72%)
No	1 (11%)	2 (22%)	2 (25%)	3 (16%)	2 (20%)	1 (17%)	11 (18%)
Not reported	1 (11%)	1 (11%)	1 (13%)	2 (11%)	0	1 (17%)	6 (10%)

**Table 5:** Significance level of (a) type I error rate and (b) CIs for all articles by whether CI was one-sided or two-sided

	One-sided	Two-sided	Not reported
(a) Type I error rate (%)			
0.8	0	1 (1%)	0
1.25	3 (2)	0	0
2.45	1 (1)	0	0
2.5	40 (24)	2 (1)	2 (1)
5	46 (27)	29 (17)	15 (9)
10	1 (1)	2 (1)	0
Not reported	3 (2)	0	23 (14)
(b) Significance level of CI (%)			
90	1 (1)	14 (8)	1 (1)
95	14 (8)	125 (74)	0
97.5	4 (2)	7 (4)	0
Other	0	1 (1)	0
Not reported	0	0	1 (1)

### Missing data and sensitivity analyses

Ninety-nine (59%) trials did not report whether or not any imputation was carried out and only 12 (7%) explicitly declared that no imputation was used. Assuming a worst-case scenario or multiple imputation were the most common methods used (table 6). The number of imputations used for multiple imputation was specified in 8 of 11 articles and 4 of 11 stated at least one of the assumptions from Rubin's rules.^22^ Sixty-four (38%) trials reported using sensitivity analyses to test robustness of conclusions of the primary outcome; of these, 27 (42%) were related to assumptions about the missing data (table 6).

**Table 6:** Reporting of (a) missing data and (b) sensitivity analyses

	n (%)
(a) Imputation performed	
Yes	56 (33)
Worst-case scenario	19 (34)
Multiple imputation	11 (20)
Last observation carried forward	8 (14)
Complete case analysis	6 (11)
Best-case scenario	2 (4)
Last observation carried forward and worst-case scenario	2 (4)
Best-case/worst-case scenario	3 (5)
Mean imputation	1 (2)
Complete case analysis, multiple imputation using propensity scores and multiple imputation using regression modelling	1 (2)
Other and worst-case scenario	1 (2)
Other	1 (2)
No	12 (7)
Not reported	99 (59)
Unclear	1 (1)
Including *NEJM* protocols (N=61)	
Yes	22 (36)
No	7 (11)
Not reported	31 (51)
Unclear	1 (2)
(b) Sensitivity analyses performed	
Yes	64 (38)
Patient population	13 (20)
Competing risks	2 (3)
Statistical modelling	2 (3)
Adjusted for baseline variables	1 (2)
Excluded protocol violations	1 (2)
On-treatment	1 (2)
Patient population/other	1 (2)
Unclear	2 (3)
Other	15 (23)
Missing data	27 (42)
Best-case/worst-case scenario	5
Complete case analysis	3
Imputation of missing values	3
Multiple imputation	3
Worst-case scenario	3
Baseline observation carried forward	1
Baseline observation carried forward and complete case analysis	1
Complete case analysis, multiple imputation using propensity scores and multiple imputation using regression modelling	1
Complete case analysis and missing not at random	1
Complete case analysis and best-case scenario	1
Different methods	1
Last observation carried forward	1
Modelling	1
Observed failure	1
Worst-case scenario and last observation carried forward	1
No	103 (61)
Unclear	1 (1)
Including *NEJM* protocols	
Yes	38 (62)
No	23 (38)

### Study conclusions

There were seven (4%) articles that could not make definitive conclusions (noted as ‘other’; table 7). For example, if all analyses conducted had to demonstrate non-inferiority to conclude a treatment was non-inferior, and only one of the analyses did, then non-inferiority could not be concluded and could not be rejected. Non-inferiority was declared in 132 (79%) articles. Ten of these had made some reference with equivalence studies within the article (see online supplementary material).

Superiority analyses were performed in 37 (22%) trials after declaring non-inferiority; of which, 27 (73%) had explicitly preplanned for superiority analyses. p Values were reported in 98 (58%) articles; of which, 29 (30%) were testing a superiority hypothesis.

### Subgroup of trials with published protocols

Additional information from protocols published by *NEJM* was extracted for 57 of 61 articles. Including this additional information provided by *NEJM* improved reporting of results across all criteria: 39 (64%) articles justified the choice of the non-inferiority margin compared to 19 (31%); most planned two or more analyses 45 (74%) compared to 37 (61%) (there were a couple of cases where two analyses were planned in the protocol but only one was stated in the published article); consistency between type I error rates and CIs was 44 (72%) compared with 36 (59%); imputation techniques were considered in 29 (48%) compared with 17 (28%) articles and sensitivity analyses were considered in 38 (62%) articles compared with 25 (41%). The majority of articles concluded non-inferiority with 8 (13%) not determining non-inferiority. A total of 14 (23%) articles concluded superiority, of which most were pre-planned (9; 64%). Few articles 8/40 (20%) presented superiority p values.

### Association between quality of reporting and conclusions

Trials that were classed as having some ‘other’ conclusion about non-inferiority were excluded from the analysis. Overall, there was a suggestive difference between the quality of reporting and concluding non-inferiority: 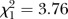; p=0.05 (Cochran–Armitage test; table 7). Trials that were poorly reported were less likely to conclude non-inferiority than those that satisfied two or all criteria from justifying the choice of the margin, reporting two or more analyses or reporting a CI consistent with the type I error rate.

**Table 7:** Quality of reporting of trials associated with conclusions of non-inferiority

	Concluded non-inferiority			
	Yes (N=132)	No (N=29)	Other (N=7)	Total (N=168)
Grade	n (%)	n (%)	n (%)	n (%)
Excellent†	11 (73)	2 (13)	2 (13)	15
Good‡	55 (86)	9 (14)	0 (0)	64
Fair§	48 (80)	8 (13)	4 (7)	60
Poor¶	18 (62)	10 (34)	1 (3)	29

*Excluding trials that concluded ‘other’: 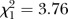; p=0.05 (Cochran–Armitage test).

†Excellent if margin justified, ≥2 analyses on patient population performed, type I error rate consistent with significance level of CI.

‡Good if fulfilled two of the following: margin justified, ≥2 analyses on patient population performed, type I error rate consistent with significance level of CI.

§Fair if fulfilled one of the following: margin justified, ≥2 analyses on patient population performed, type I error rate consistent with significance level of CI.

¶Poor if margin not justified, <2 analyses on patient population performed, type I error rate not consistent with significance level of CI.

## Discussion

Reporting of non-inferiority trials is poor and is perhaps partly due to disagreement between guidelines on vital issues. There are some aspects that guidelines agree on, such as a requirement for the non-inferiority margin to be justified, but we find that this recommendation is neglected by the majority of authors. It is remarkable that several authors performed only one analysis for the primary outcome and the lack of consistency between the significance level chosen in sample size calculations and the CI reported further highlights confusion of non-inferiority trials. Not knowing how to deal with missing data nor appropriate sensitivity analyses, also adds to the confusion. The combination of these recent findings assessed from high-impact journals and the inconsistency in guidelines indicate: (1) the non-inferiority design is not well understood by those using the design and (2) methods for non-inferiority designs are yet to be optimised.

We anticipated that poor reporting of articles would bias towards concluding non-inferiority; however, the poorly reported trials were less likely to demonstrate non-inferiority. This is somewhat reassuring. Nevertheless, it is essential to ensure that what is reported at the end of a trial was prespecified before the start of a trial: scientific credibility and regulatory acceptability of a non-inferiority trial rely on the trial being well-designed and conducted according to the design.^23^ It is possible that the quality of a trial may also depend on the quality of the outcome; unresponsive outcomes that miss important differences between treatments may be intentionally or unintentionally chosen to demonstrate non-inferiority. Therefore, it is also important that the outcome chosen is robust.

Almost 80% of studies concluded non-inferiority, although it is unclear whether this is due to the reporting in articles or publication bias. It appears that positive results (ie, alternative hypotheses) are published more often, regardless of trial design, as this number is consistent with other studies that found that more than 70% of published superiority trials demonstrated superiority.^24^ ^25^

More than half of articles reported p values, of which approximately a third reported p values for a two-sided test for superiority. p Values, if reported, should be calculated for one-sided tests corresponding to the non-inferiority hypothesis; that is, with H_0_: δ=margin. p Values for superiority should not be presented unless following the demonstration of non-inferiority, where a preplanned superiority hypothesis is tested.^26^

### Comparison with other studies

The value of the non-inferiority margin was almost always reported, but more than half of articles made no attempt to explain how the choice was justified. While justification of the margin is low, this is actually an improvement from Schiller *et al*^27^ who reported 23% articles made a justification, although this difference could be because only high-impact journals were included in this review. There were equally as many articles that planned and reported an ITT analysis compared with articles that performed ITT and PP analyses. This is surprising given that CONSORT 2006 states that an ITT analysis can bias non-inferiority trials towards showing non-inferiority.^1^ These results were lower than found by Wangge *et al*^28^ who reported 55% used either an ITT or PP and 42% used ITT and PP. Most articles presented two-sided 95% CIs, which is consistent with results from Le Henanff *et al*.^29^

There were very few articles that referred to preserving the treatment effect based on estimates of the standard of care arm from previous trials. It is vital that authors acknowledge this to ensure the standard of care is effective. If the control was to have no effect at all in the study, then finding a small difference between the standard of care and new intervention would be meaningless.^2^

Clinical considerations^1^ ^2^ ^9^ ^11–13^ to justify the choice of the margin often had inadequate justifications, such as ‘deemed appropriate’ or ‘consensus among a group of clinical experts’. Non-inferiority is only meaningful if it has strong justification in the clinical context and so should be reported. If the justification includes a measurable reduction in adverse events, these should be measured and the benefit should be demonstrated. Guidelines recommend that the choice of margin should be justified primarily on clinical grounds; however, previous trials and historical data should also be considered if available. As an example, Gallagher *et al*^30^ justify the choice of the margin providing as much information as possible by including references to all published reports and providing data from the institution where the senior author is based.

A statement often used in articles reviewed was ‘the choice of the margin was clinically acceptable’. This statement does not contain enough information to justify the choice of the non-inferiority margin. If the choice of the margin is based on a group of clinical experts, authors should provide information on how many experts were involved and how many considered the choice of the margin being acceptable: a consensus among a group of 3 clinicians from 1 institution is different from a consensus of 20 clinicians representing several institutions. Radford *et al*^31^ justify the choice of the non-inferiority margin after performing a delegate survey at a symposium. This method may be a way forward for researchers to obtain clinical assessment from a large group of clinicians. Even better would be to obtain formal assessments, using, for example, the Delphi method,^32^ which has been used in the COMET initiative,^33^ after presenting the proposed research at a conference or symposium for clinicians to really engage with the question at hand.

Definitions provided by authors were inconsistent under what they classed as ITT, PP, mITT and as-treated, for example, “all patients randomised who received at least one dose of treatment” was defined at least once in each classification. According to the guidelines, the PP definition excludes patients from the analysis, but it is unclear what those exclusions are. The ambiguity of how PP is defined was evident in this review as definitions provided by authors could not be succinctly categorised.

Many articles presented only one analysis, despite most guidelines recommending at least two analyses.^1^ ^2^ ^9^ ^10^ ^12^ Unfortunately, guidelines differ in their advice on which of the two analyses should be chosen to base conclusions on. This regrettable, state of affairs was clearly reflected in our review.

The ITT and PP analyses have their biases and so neither can be taken as a ‘gold standard’ for non-inferiority trials. The analysis of the primary outcome is the most important result for any clinical trial. It should be predefined in the protocol what patients should adhere to and should be considered at the design stage what can be carried out to maximise adherence. It should be made clear exactly who is included in analyses given the variety of definitions provided by various authors, particularly for PP analyses where definitions are subjective. Most authors included treatment-related exclusions such as ‘received treatment’, ‘completed treatment’ or ‘received the correct treatment’. Such differences in definitions may be superficially small, but could in fact make critical differences to the results of a trial.

Poor reporting of whether the hypothesis test was one-sided or two-sided or absence of the type I error rate in the sample size calculation meant over a quarter of articles were not clearly consistent with regard to the type I error rate and corresponding CI.

Most guidelines advise presenting two-sided 95% CIs and this is what most articles presented. However, this recommendation may cause some confusion between equivalence and non-inferiority trials. A 5% significance level is maintained using 95% CIs in equivalence trials for two-sided hypotheses, whereas non-inferiority takes a one-sided hypothesis and so a two-sided 90% CI should be calculated. If a one-sided type I error rate of 2.5% is used in the sample size calculation, then this corresponds to the stricter two-sided 95% CIs, not a one-sided 95% CI.^34^

The power and type I error rate should be clearly reported within sample size calculations and whether the type I error rate is for a one-sided or two-sided test. For example, the CAP-START trial used a one-sided significance test of 0.05 with two-sided 90% CIs, and the authors provide exact details of the sample size calculation in online supplementary appendix.^35^ If presenting one bound of the CI throughout an article, this must be performed clearly and consistently as described by Schulz-Schüpke *et al*,^36^ Lucas *et al*,^37^ Gülmezoglu *et al*.^38^ Recently, *JAMA* have introduced a policy to present the lower bound of the CI with the upper bound tending towards infinity,^39^ and this has been put into practice in recent non-inferiority trials.^40–43^

It is unclear whether the potential issues surrounding missing data are well recognised for non-inferiority studies, given that the majority of articles did not explicitly state whether or not methods to handle missing outcome data would be considered. Most trials that used multiple imputation stated the number of imputations used but few discussed the assumptions made, which are particularly critical in this context. Some missing data are inevitable, but naive assumptions and/or analysis threaten trial validity for ITT and PP analyses,^14^ particularly in the non-inferiority context where more missing data can bias towards demonstrating non-inferiority.^44^

It is recommended for trials to clearly report whether imputation methods to handle missing data were or were not performed. If imputation was used, it should be clearly stated what method was used along with any assumptions made, following the guidelines of Sterne *et al*.^45^

Only about a third of articles reviewed reported using sensitivity analyses. There was some confusion between sensitivity analyses for missing data and secondary analyses. Sensitivity analyses for missing data should keep the primary analysis model, but vary the assumptions about the distribution of the missing data, to establish the robustness of inference for the primary analysis to the inevitably untestable assumptions about the missing data. In contrast, secondary analysis with regard to excluding patients for the primary outcome is attempting to answer a separate, secondary question.^46^ Thus, while EMEA 2000 and CONSORT 2012 describe this as sensitivity analysis (and many papers we reviewed followed this), in general this will not be the case, and conflating the two inevitably leads to further confusion.

The focus of the analysis for non-inferiority trials should be on patients who behaved as they were supposed to within a trial, that is the PP population, but rather than excluding patients from the PP analyses, an alternative approach would be to make an assumption about the missing data for patients who do not adhere to the predefined PP definition and then impute missing outcomes for these patients as if they had continued in the trial without deviating. Sensitivity analyses should then be used to check robustness of these results. However, currently, it is unclear what methods are appropriate to achieve this goal.

### Subgroup of trials with published protocols

The mandatory publication of protocols taken from *NEJM* publications improved results for all criteria assessed. This reiterates the findings from Vale *et al*^47^ who evaluated the risk of bias assessments in systematic reviews assessed from published reports, but had also accessed protocols directly from the trial investigators and found that deficiencies in the medical journal reports of trials does not necessarily reflect deficiencies in trial quality. Given this, it is clear that a major improvement in the reporting of non-inferiority trials would result if all journals followed the practice. Since publication of e-supplements is very cheap, there appears to be no reason not to do this.

### Strengths and limitations

This research demonstrates the inconsistency in the recommendations for non-inferiority trials provided by the available guidelines, which was also reflected within this review. We have provided several recommendations using examples for researchers wishing to use the non-inferiority design and have outlined the most important recommendations that we hope will be taken up in future guidelines (box 1). We have also highlighted the importance of missing data and using sensitivity analyses specific to non-inferiority trials. There are also some limitations in this review. First, a justification of the choice of the margin was recorded as such if any attempt was made to do so. Therefore, one could argue that inadequate attempts were counted as a ‘justification’; however, there was good agreement between reviewers when independently assessed. Second, only one reviewer extracted information from all articles and therefore assessments may be subjective. However, there was good agreement when a random 5% of papers were independently assessed, and the categorisation of the justification of the non-inferiority margin was also independently assessed in all papers where a justification was given. Third, an update of the CONSORT statement for non-inferiority trials was published during the period of the search in 2012,^9^ which could improve the reporting of non-inferiority trials over the next few years. However, the first CONSORT statement for non-inferiority trials published in 2006^1^ was released well before the studies included in our search and we have found that reporting of non-inferiority trials remains poor.

Box 1 **Recommendations**

▸ Justification of the margin should be a made mandatory in journals.

▸ Authors should make reference to preserving the treatment effect based on estimates of the standard-of-care arm from previous trials.

▸ Presentation of the CI should be consistent with the type I error rate used in sample size calculations.

▸ Analyses should be performed to answer the question of interest (ie, the primary outcome) using additional analyses to test the robustness of that definition, rather than to heedlessly satisfy intention-to-treat and per-protocol definitions.

▸ Methods to handle missing data should be considered, and sensitivity analyses should be considered to test the assumptions of missing data made on the primary analysis.

▸ Protocols should always be published as online supplementary material and authors should make use of online supplementary material to include additional detail on methods (such as details for justifying the choice of the non-inferiority margin and full definition of analyses conducted), so that a word limit for a published article should not be an excuse for poor reporting.

## Conclusion

Our findings suggest clear violations of available guidelines, including the CONSORT 2006 statement (published 4 years before the first paper in our review), which concentrate on improving how non-inferiority trials are reported and is widely endorsed across medical journals.

There is some indication that the quality of reporting for non-inferiority studies can affect the conclusions made and therefore the results of trials that fail to clearly report the items discussed above should be interpreted cautiously. It is essential that justification for the choice of the non-inferiority margin becomes standard practice, providing the information early on when planning a study including as much detail as possible. If the choice of the non-inferiority margin changes following approval from an ethics committee, justification for the change and changes to the original sample size calculation should be explicit. If journals enforced a policy where authors must justify the choice of the non-inferiority margin prior to accepting publication, this would encourage authors to provide robust justifications for something so critical given that clinical practice may be expected to change if the margin of non-inferiority is met.

Sample size calculations include consideration of the type I error rate, which should be consistent with the CIs as these provide inferences made for non-inferiority when compared against the margin. Inconsistency between the two may distort inferences made, and stricter CIs may lack power to detect true differences for the original sample size calculation. If any imputation was performed, then this should be detailed along with its underlying assumptions, supplemented with sensitivity analyses under different assumptions about the missing data. There is an urgent need for research into appropriate ways of handling missing data in the PP analysis for non-inferiority trials; once resolved, this analysis should be the primary analysis.

Information that is partially prespecified before the conduct of a trial may inadvertently provide opportunities to modify decisions that were not prespecified at the time of reporting without providing any justification. It is therefore crucial for editors to be satisfied that criteria are defined a priori. A compulsory requirement from journals to publish protocols as e-supplements and even statistical analysis plans along with the main article would avoid this ambiguity.
