# Supplementary material for: Non-inferiority trials: are they inferior? A systematic review of reporting in major medical journals
Source: BMJ Open. 2016 Oct 7;6(10):e012594. doi: 10.1136/bmjopen-2016-012594 (PMC5073571; doi:10.1136/bmjopen-2016-012594)
Supplement: Supplementary data [file bmjopen-2016-012594supp.pdf]

Supplement

Methods

Data extraction form

The form was tested by two reviewers (SR & TM) on articles, included in this review, until agreement was achieved between both reviewers. Justifications for the choice of the non-inferiority margin were reviewed by two reviewers due to its complexity. The power from the planned sample size calculation was recorded from the methods section. We recorded what analyses was used for the primary outcome and we noted how this was defined according to authors. This was either extracted from the main text or from the CONSORT flow chart. Definitions that were provided but not classed as ITT, PP, mITT or as-treated were categorised accordingly.

Definition of patient population

If definitions were provided on what patient population was included in analyses but were not classed by authors, then the definitions were categorised as follows:

- All patients randomised into the study were analysed was classed as an *intention-to-treat* analysis
- Patients who were excluded after administration of treatment (e.g. withdrawals, loss to follow up, compliance) was classed as a *per-protocol* analysis
- Patients who were excluded after administration of treatment, but the exclusion was not treatment related (e.g. patients who did not have the disease of interest) was classed as a *modified intention-to-treat* analysis
- Analysis based on what treatment patients actually received as opposed to the treatment that was allocated at the time of randomisation was classed as an *as-treated* analysis

Determining whether the analysis of the patient population was primary or secondary

Information on whether a patient population was considered as a primary analysis or secondary analysis (for the same primary outcome) was collected. The population was assumed primary if only one analysis was reported. If more than one analysis was performed but it was not clearly described which was to be taken as the primary and/or secondary analysis, the primary analysis was assumed to be whatever was presented in the results section of the abstract and secondary if not presented in the abstract but stated elsewhere within the article. If all results were presented for all populations in the abstract, then both were assumed as primary unless non-inferiority was concluded on only one patient population. Analysis was assumed secondary if the patient population was stated but not defined or if the results of the analysis were not presented in the article.

Results

Reasons for "Other" justification of non-inferiority margin

For all articles

There were 12(7%) justifications classed as "other":

- Based on previous trial. No evidence for consultation with external expert group, and no reference to previous trial of the control arm
- Based on unpublished data. No evidence for consultation with external expert group, and no reference to previous trials of the control arm
- Clinical basis and based on previous trials and guidelines. No evidence for consultation with external expert group, and no reference to previous trials of the control arm
- Clinical basis. Attempted to justify based on preservation of treatment effect, but were unable to do so due to paucity of previous trials.
- Expert group external to the authors and previous trial. No reference to previous trial of the control arm
- Justified based on treatment effect of control, but margin actually bigger than control arm treatment effect
- Placebo controlled study. Clinical basis, previous trials and literature review
- Preservation of treatment effect. Reference to separate paper justifying margin
- Regulatory guidelines (WHO), but recommendation is for superiority. No evidence for consultation with external expert group, and no reference to previous trials of the control arm
- Synthesis approach
- Unclear

NEJM protocols

There were 6 (10%) justifications classed as "other":

- Based on previous trial. No evidence for consultation with external expert group, and no reference to previous trial of the control arm
- General comment that margin was decided according to FDA request
- Justified based on treatment effect of control, but margin actually bigger than control arm treatment effect
- Preservation of treatment effect based on estimates of control arm effect from previous trials and clinical basis
- Preservation of treatment effect based on estimates of control arm effect from previous trials, clinical basis and according to FDA guidelines
- Preservation of treatment effect. Reference to separate paper justifying margin

Table 1a: Type of analysis chosen

	All articles	NEJM protocols
**Analysis**	**n (%)**	**n (%)**
ITT only	54 (32%)	12 (20%)
PP only	3 (2%)	0
mITT only	8 (5%)	3 (5%)
ITT and PP	56 (33%)	17 (28%)
ITT and mITT	3 (2%)	2 (3%)
ITT and as-treated	4 (2%)	4 (7%)
ITT and other definition	6 (4%)	2 (3%)
PP and mITT	17 (10%)	9 (15%)
PP and other definition	4 (2%)	2 (3%)
mITT and as-treated	0 (0%)	1 (2%)
mITT and other definition	1 (1%)	1 (2%)
ITT, PP and mITT	1 (1%)	1 (2%)
ITT, PP and as-treated	0 (0%)	1 (2%)
ITT, PP and other definition	5 (3%)	5 (8%)
mITT, PP and other definition	4 (2%)	0
Unclear	2 (1%)	1 (2%)

Figure 1a: Chosen analysis by primary or secondary analysis

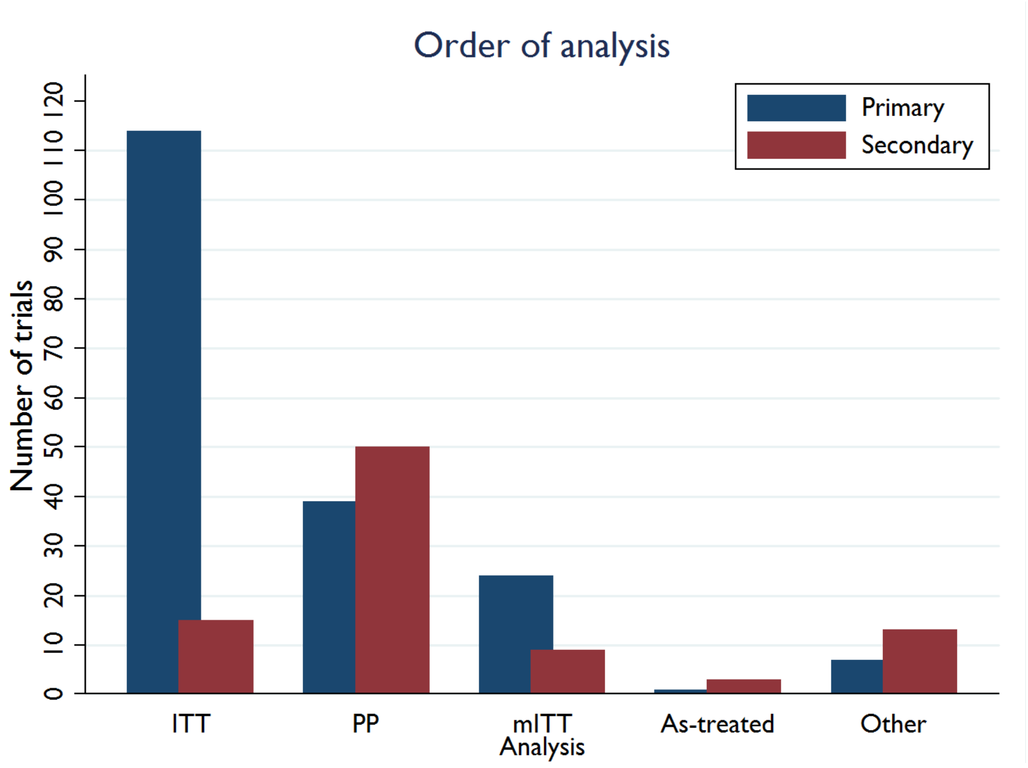

NB: One study performed ITT and PP analyses but it was unclear which of the two was taken as primary and secondary

Table 1b: Definition of analysis

Analysis	Definition	n (%)
**ITT**		**129**
	All patients randomised	68 (53%)
	all patients randomised who received at least one dose of treatment/intervention	21 (16%)
	All patients randomised excluding missing data	7 (5%)
	All patients randomised excluding errors in randomisation	3 (2%)
	All patients randomised who received at least one dose of treatment/intervention, excluding missing data	1 (1%)
	All patients randomised with exclusions from one centre which was removed due to misconduct	1 (1%)
	Other	17 (13%)
	Unclear	1 (1%)
	Not defined	10 (8%)
**PP**		**90**
	Patients who received allocated treatment/intervention	8 (9%)
	Excluding patients with major protocol violations	5 (6%)
	Patients who completed allocated treatment/intervention as intended	4 (4%)
	Patients who adhered to treatment	2 (2%)
	Excluding patients with protocol deviations	2 (2%)
	Patients with no exclusion criteria and who received specific amount of treatment/intervention	2 (2%)
	Patients who received allocated treatment/intervention, no major protocol violations with outcome	2 (2%)
	Excluding patients who switched treatment	1 (1%)
	Patients who received at least one dose of treatment/intervention	1 (1%)
	Patients who adhered to the protocol	1 (1%)
	Patients who completed the assigned study regimen or adhered to treatment before an event	1 (1%)
	Patients who received correctly allocated treatment/intervention excluding withdrawals	1 (1%)
	Patients who received specific amount of treatment/intervention and adhered to protocol	1 (1%)
	Patients who received allocated treatment/intervention, excluding non-adherence	1 (1%)
	Patients who adhered to protocol excluding withdrawals	1 (1%)
	Excluded patients with protocol deviations in addition to mITT definition	1 (1%)
	excluded patients that received rescue medication and protocol violations	1 (1%)
	Patients who received at least one dose of drug/intervention and received allocated treatment/intervention excluding missing outcome data	1 (1%)
	All patients who received at least one dose of treatment/intervention and did not have major protocol violations and were followed for event while receiving drug	1 (1%)

	All patients who received at least one dose of treatment/intervention and did not have major protocol violations	1 (1%)
	Excluding patients who were ineligibile, excluding patients who were administered the incorrect dose of medication and excluding patients who were allocated the incorrect treatment	1 (1%)
	All patients randomised who received at least one dose of treatment/intervention with an outcome, completed the study and complied with protocol	1 (1%)
	Non-adherence, patients who declined follow up, errors in randomisation, recurrent atrial fibrillation before randomisation were excluded	1 (1%)
	The per-protocol population (which consisted of the modified intention-to-treat population with the exclusion of patients with major protocol deviations and a compliance rate of <80%) was of primary interest, since a noninferiority analysis that is based on the modified intention-to-treat population is deemed to be not conservative	1 (1%)
	Patients were not eligible for per-protocol analysis for the following reasons: no follow-up visit; systemic treatment with other antimicrobial drugs up to day 28 (visit three); or missing more than one dose of the study drug during the first week of treatment or more than two doses during the whole treatment period	1 (1%)
	Excluded missing inclusion criteria; incorrect dosing; received prohibited medication; missing assessments	1 (1%)
	Per-protocol analyses excluded participants who had missing data at 1 month or who had major protocol violations (e.g., death, pregnancy, withdrawal from the study, loss to follow-up, or noncompliance).<br>NB: Two results were presented for PP where compliance was included and excluded.	1 (1%)
	Per-protocol prespecified analyses included children with complete follow-up or a confirmed treatment failure, and excluded those treated for malaria without confirmatory microscopy, those for whom the alternative Plasmodium species was detected, and those who defaulted from follow-up despite repeated attempts at contact<br>Flow chart includes: “and followed protocol”	1 (1%)
	Patients who, during the intended treatment period, had a venogram adjudicated as assessable, who developed confirmed deep vein thrombosis or pulmonary embolism, or who died from any cause); patients who had important protocol violations were excluded from the per-protocol analysis.	1 (1%)
	The per-protocol population was defined as all patients included in the ITT analysis, excluding those who did not receive the regimen as prescribed. These were patients who received less than 6 weeks of treatment (42 days of daily treatment or 36 days of 6-days-a-week treatment) or more than 9 weeks of treatment (63 days of daily treatment or 54 days of 6-days-a-week treatment) in the intensive phase and those who received less than 42 doses (ie, 4 weeks of missed treatment) or more than 60 doses (ie, 2 weeks of extra treatment) in the continuation phase (the protocol requirement is that patients receive 18 weeks of 3- times-weekly treatment, ie, 54 doses). Also excluded were patients whose treatment was modified for reasons other than bacteriological failure or relapse (including patients changing treatment for adverse drug reactions, following return after default, or attributable to concomitant HIV infection).	1 (1%)
	Per-protocol snapshot analysis, which included all participants who were enrolled, received at least one dose of study drug, and did not meet any of the following prespecifi ed criteria: discontinuation of study drug before week 48 or HIV RNA data missing in week 48 analysis window (accounting for 80% of excluded patients), and adherence in the bottom 2.5th percentile (accounting for 20% of the excluded patients)	1 (1%)
	The perprotocol group consisted of all patients who were enrolled, had no major protocol deviation, received the full treatment, and were assessed at day 15 or 31,	1 (1%)

	day 45, and 6 months (-2 to +6 weeks).	
	Criteria to exclude patients from this set were violation of major in- or exclusion criteria, change of treatment arm, early treatment discontinuation or relevant dose deviations of chemo- or radiotherapy unless caused by death or progression, radiotherapy without PET panel recommendation or omission of radiotherapy against recommendation, PET panel decision to take the patient off protocol treatment, or missing documentation of treatment	1 (1%)
	The per-protocol analysis set additionally excludes patients with change of treatment arm, early treatment discontinuation or relevant dose deviations of chemo- or radiotherapy unless caused by death or progression, or missing documentation of treatment	1 (1%)
	The perprotocol analysis was based on all participants who received 3 doses of vaccine according to 1 of the study's vaccine dosing schedules, were seronegative to the relevant HPV type at baseline, and had a valid serology result after the third dose of the HPV vaccine	1 (1%)
	Not defined. Taken from flow chart: Patients not meeting the definition of having received adequate treatment provided they have not already had an unfavourable response to treatment. Other exclusions done as well, but are not defined in flow chart	1 (1%)
	All patients who underwent randomization, completed a full treatment course or had early treatment failure before treatment was completed, had outcome data for the primary efficacy end point on day 28, and complied with the protocol to the extent that would allow efficacy evaluation	1 (1%)
	We also conducted a perprotocol analysis, which included those who completed the 2-month visit while receiving treatment (108 oral, 113 intratympanic) because intention-to-treat analyses may bias toward noninferiority. Flow chart also shows patients who withdrew before the 2m follow up, those who discontinued treatment but completed follow up and those who completed treatment but missed 2m follow up were excluded.	1 (1%)
	Which consisted of participants who received all three doses of vaccine within 1 year, did not have the HPV type being analyzed (i.e., were seronegative on day 1 and PCR-negative from day 1 through month 7), and had no protocol violations	1 (1%)
	A total of 12 (10%) patients in each group did not undergo PEG for anatomical reasons. Between the PEG procedure and the follow-up visit, five patients died, one patient pulled out the PEG catheter without ensuing complications, three patients were lost to follow-up, and one patient who was randomised to cefuroxime received co-trimoxazole instead.	1 (1%)
	Will include all subjects in the MITT population grouped by randomized treatment assignment regardless of treatment received with the exception of the following additional exclusions<br>1. Subjects not meeting the definition of having received an adequate amount of their allocated study regimen (see below for definition), provided they have not already been classified as having an unfavourable outcome<br>2. Subjects lost to follow-up or withdrawn before the Month 6 visit, unless they have already been classified as having an unfavourable outcome.<br>3. Subjects whose treatment was modified or extended for reasons (e.g. an adverse drug reaction or pregnancy) other than an unfavourable therapeutic response to treatment, unless they have already been classified as having an unfavourable outcome<br>4. Subjects who are classified as "major protocol violations" (see section 6.5), unless they have already been classified as having an unfavourable outcome on the basis of data obtained prior to the protocol violation	1 (1%)
	The per-protocol analysis excluding the 6 patients who were lost to follow-up and the 3 patients who received postoperative corticosteroids (including the 4 patients who experienced primary bleeding events)	1 (1%)

	Excluded patients who received a platelet transfusion for reasons not recommended in the protocol	1 (1%)
	We also did a per-protocol analysis of the medical outcomes, excluding outpatients discharged more than 24 h after randomisation and inpatients discharged 24 h or less after randomisation.	1 (1%)
	The perprotocol population was defined as intention-to-treat patients with (1) successful procedure outcome, (2) treatment solely with the zotarolimus-eluting stent, (3) dual antiplatelet therapy according to randomization, and (4) complete clinical follow-up information.	1 (1%)
	Not defined. Flow chart shows the following exclusions: had another histology or malignancy; withdrew informed consent; had an allergic reaction on first rituximab infusion and consecutively other treatment; only had radiotherapy; received incorrectly allocated treatment; did not meet inclusion or exclusion criteria; no therapy; death before therapy	1 (1%)
	Not defined. Flow chart suggests patients were excluded if they did not receive the protocol and withdrawals	1 (1%)
	Censoring of events if any component of the initial randomised trial treatment was stopped	1 (1%)
	Not defined. Flow chart shows inclusion/exclusion criteria violated, non-adherence, prohibited medication and missing results were excluded	1 (1%)
	Participants who did not follow protocol and/or were seropositive or polymerase chain reaction-positive for HPV-16, HPV- 18, HPV-6, or HPV-11 at enrolment were excluded from the per-protocol population analysis but retained for the intention-to-treat population analysis. Participants were eligible to continue with the 18- and 36-month follow-up if they had all of their doses of vaccine and a 7-month blood sample collected. If participants were excluded from the per-protocol population analysis at 7 months, they remained excluded for the remainder of the study but were retained for intention- to-treat analysis.	1 (1%)
	The per-protocol population included all patients who completed the study (1 year), and for whom the second reading of a CT-scan confirmed the diagnosis of uncomplicated appendicitis.	1 (1%)
	For analyses based on the per-protocol population, patients were analysed according to their randomly assigned treatment group. To be included in the perprotocol population, a patient was required to meet the following criteria: Had a mean baseline hemoglobin ≥ 8.0 and < 11.0 g/dl; Completed the study through at least week 36, and at least 5 hemoglobin values were obtained during the evaluation period; Had no missing administrations of study medication between weeks 21 and 35, inclusive; Had not received any RBC or whole blood transfusions within the 12 weeks prior to randomization; Had not received any RBC or whole blood transfusions for reasons other than lack of effect of study medication (lack of effect of study medication was documented as “Anemia of CRF” on the case report form) between weeks 21 and 35, inclusive; Had not received any ESA other than the assigned study treatment between weeks 21 and 35, inclusive; Had adequate iron status at baseline and during the evaluation period (defined as serum ferritin ≥100 ng/ml and TSAT ≥ 20% during weeks 24, 28, and 32)	1 (1%)
	Not defined. Flow chart shows exclusions: caesarean section or forceps; short umbilical cord or nuchal cord; need for resuscitation; team became unavailable; weight scale malfunctioned; parent withdrew consent	1 (1%)
	Completers (observed cases; included patients in the full analysis set who did not have important protocol violations, completed at least 684 days of treatment, and had HbA1c measured at week 104)	1 (1%)
	For analyses based on the per-protocol population, patients were analyzed according to their randomly assigned treatment group. To be included in the per-protocol population, a patient was required to meet the following criteria: Had a mean baseline hemoglobin ≥ 10.0 and ≤ 12.0 g/dl; Completed the study through at least week 36, and at least six haemoglobin values were obtained during the	1 (1%)

	evaluation period.; Received ≥ 75% of total prescribed (i.e., expected) doses of study medication between weeks 25 and 35, inclusive (detailed algorithms for this determination were specified in the Statistical Analysis Plan).; Had not received any RBC transfusions within the 12 weeks prior to randomization.; Had not received any RBC transfusions for reasons other than lack of effect of study medication (lack of effect of study medication was documented as “Anemia of CRF” on the case report form) between weeks 25 and 36, inclusive.; Had not received any ESA other than the assigned study treatment between weeks 25 and 35, inclusive.; Had adequate iron status at baseline and at week 36 (defined as serum ferritin ≥ 100 ng/ml and TSAT ≥ 20%).	
	This population included all patients who underwent randomisation and who completed the study procedures to month 6.	1 (1%)
	We also performed a per-protocol analysis, which notably excluded patients in the antibiotic group who had been switched from amoxicillin plus clavulanic acid to another antibiotic.	1 (1%)
	We did a per-protocol snapshot analysis, which included all participants who were randomly assigned treatment, received at least one dose of study drug, and did not meet any of the following prespecifi ed criteria: discontinuation of study drug before week 48 or HIV RNA results missing in the week 48 analysis window, and adherence in the bottom 2.5th percentile.	1 (1%)
	Patients were included in the per-protocol population if they met the criteria for inclusion in the modified intention-to-treat population, underwent an adequate assessment of venous thromboembolism not later than 2 days after administration of the last dose of study drug, and had no major protocol violations.	1 (1%)
	The perprotocol population comprised patients in the modified intention-to-treat group who received treatment for at least 3 days (in the case of patients with treatment failure) or at least 8 days (in the case of patients with clinical cure), had documented adherence to the protocol, and underwent an end-of-therapy evaluation.	1 (1%)
	The per-protocol analysis set consisted of participants with exposure to treatment for at least 12 weeks who did not have any major protocol violations that could affect the primary endpoint and had a valid glycated haemoglobin (HbA1c) assessment at baseline and at (or after) 12 weeks.	1 (1%)
	Not defined	11 (12%)
**mITT**		**34**
	All patients randomised who received at least one dose of treatment/intervention	10 (29%)
	All patients randomised who received at least one dose of treatment/intervention, excluding missing data	6 (18%)
	All patients randomised with at least one dose of treatment/intervention excluding patients/site with violations of GCP	2 (6%)
	All randomised patients who received at least one dose of treatment/intervention excluding patients without disease or excluding patients resistant to one of the drug combinations. Excluding patients whose death was not related to the disease or had reinfection after being cured or patients who were classed as unassessable at the endpoint	1 (3%)
	Patients were excluded if they were resistant to two of the treatment combinations and patients who were unassessable and had not reached endpoint	1 (3%)
	On-treatment which included events that occurred within 30 days after the last dose of study medication was administered	1 (3%)
	Patients were excluded if they had missing/contaminated outcome data or could not produce an assessment or were lost to follow up or had death not related to disease or had confirmed reinfection	1 (3%)

	Excluded if consent withdrawn, non-compliance, moved and other (other not defined)	1 (3%)
	Other	11 (32%)
**As-treated**		**4**
	All patients randomised who received intervention	1 (25%)
	Not defined	3 (75%)
**Other**		**20**
	Full analysis set	4 (20%)
	On treatment analysis	3 (15%)
	Complete follow up data	1 (5%)
	ITT efficacy	1 (5%)
	PP and modified PP	1 (5%)
	Should be classed as PP. All patients who completed study with no major protocol deviations	1 (5%)
	Should be classed as mITT	2 (10%)
	Should be classed as mITT (ITT with no exclusion criteria)	1 (5%)
	Should be as treated (treatment received)	1 (5%)
	Other	5 (25%)
**Unclear**		**2**

Study conclusions

Of the articles that were designed as non-inferiority trials, two articles stated the trial was non-inferiority, but had drawn equivalence graphs with two margins; one article stated the trial was for non-inferiority but states the sample size calculation is to determine equivalence; one article concluded that their study did not show equivalence; one concluded equivalence; one article stated that the margin was an equivalence margin; one stated that they would test for equivalence; one concluded non-inferiority as the confidence interval was within ±margin; one concluded equivalence in the abstract but non-inferiority in the main paper; one stated that "results were consistent with showing non-inferiority (i.e. equivalence)".
